# Imaging current distribution in a topological insulator Bi_2_Se_3_ in the presence of competing surface and bulk contributions to conductivity

**DOI:** 10.1038/s41598-021-86706-0

**Published:** 2021-04-02

**Authors:** Amit Jash, Ankit Kumar, Sayantan Ghosh, A. Bharathi, S. S. Banerjee

**Affiliations:** 1 Department of Physics, Indian Institute of Technology Kanpur, Kanpur, 208016 Uttar Pradesh India; 2 UGC-DAE Consortium for Scientific Research, Kalpakkam, 603104 India

**Keywords:** Physics, Condensed-matter physics, Topological matter, Topological insulators

## Abstract

Two-dimensional (2D) topological surface states in a three-dimensional topological insulator (TI) should produce uniform 2D surface current distribution. However, our transport current imaging studies on Bi_2_Se_3_ thin film reveal non-uniform current sheet flow at 15 K with strong edge current flow. This is consistent with other imaging studies on thin films of Bi_2_Se_3_. In contrast to strong edge current flow in thin films, in single crystal of Bi_2_Se_3_ at 15 K our current imaging studies show the presence of 3.6 nm thick uniform 2D sheet current flow. Above 70 K, this uniform 2D sheet current sheet begins to disintegrate into a spatially non-uniform flow. The flow becomes patchy with regions having high and low current density. The area fraction of the patches with high current density rapidly decreases at temperatures above 70 K, with a temperature dependence of the form $1/\left| {T - 70} \right|^{0.35}$. The temperature scale of 70 K coincides with the onset of bulk conductivity in the crystal due to electron doping by selenium vacancy clusters in Bi_2_Se_3_. Thus our results show a temperature dependent competition between surface and bulk conductivity produces a temperature dependent variation in uniformity of current flow in the topological insulator.

## Introduction

In recent times the new class of materials viz., topological insulators (TI), have been extensively investigated. The interest has been fuelled by some of the intriguing properties of TI’s materials, for example, their peculiar band structure which is characterised by a unique topological invariant index^1,2^. They possess a topologically protected bulk gapped state with conducting edge or surface state^1–6^. Any lattice distortions are incapable of destroying the topologically protected nature of the band structure in these TI materials. Theoretically, the two-dimensional (2D) TI possess a bulk gapped state while the sample edges are conducting due to the topological surface state. In contrast, the three dimensional (3D) TI materials possess topological surface^4,7–15^ states which are like uniform 2D conducting sheets enclosing a gapped bulk. Time-reversal symmetry (TRS) protects these high electrically conducting gapless surface states in TI’s. The TI possesses spin momentum locked current carrying states with opposite spins propagating in opposite directions. This suppresses back-scattering of electrons from disorder sites^2^. A characteristic feature of TI materials is that non-magnetic disorder does not affect the electrical conduction via the edge or surface states. The conducting edge/surface states exhibit Dirac-like linear energy–momentum dispersion^2^, chiral spin texture^5^ and Landau level quantization^16^. Akin to Si of the semiconductor world, Bi_2_Se_3_ is the representative material for 3D TI’s. Interpretation of bulk electrical transport measurements in 3D TI like Bi_2_Se_3_, Bi_2_Te_2_Se materials suggested that as per expectation, at low temperature there most likely exists 2D surface states^10,11^. Direct imaging of currents in the 3D TI, Bi_2_Se_3_ thin film using scanning SQUID microscopy^17^, atom-chip microscopy^18^ and scanning photo voltage measurements^19–21^, however showed the presence of one dimensional (1D) wire like currents flow along the film edges rather than 2D sheet current flow.

While most of the current imaging studies have been in high quality TI thin films, to the best of our knowledge there are none on TI single crystals. One may also note that very few imaging studies have explored the effect of non magnetic disorder on the nature of current distribution in these 3D TI materials. Typical non-magnetic defects found in 3D TI materials are, step edges of terraces on the surface of high-quality MBE grown films of TI^22^ and vacancies in the atomic lattice of the TI’s. It is known that the step edge defects locally produce a slight change in conductivity^22^, however they do not produce any appreciable temperature dependent changes in the electrical conductivity of the TI. Although, defects like vacancy in TI significantly impacts the electrical conductivity of TI. Specifically, defects like selenium (Se) vacancies present in Bi_2_Se_3_ electron dope the TI material bulk^23–26^. Thermally activated delocalization of these doped charges in the material bulk turns the insulating bulk of the TI into an electrically conducting region^23,28^. The disorder induced bulk conduction in TI bulk (which is insulating in an ideal TI) opens up an additional conduction channel which is in parallel to the topological high conduction edge or surface state channel already present in TI’s. Recent bulk mutual inductance measurements in Bi_2_Se_3_^27,28^ shows a predominance of topological surface state conductivity at low *T*, however beyond 70 K the bulk contribution grows and competes with surface conductivity. The studies show that above 70 K the bulk conductivity is of a thermally activated nature^27,28^. In view of the above issues in TI related to transformation between surface and bulk contribution to conductivity, there have been few systematic imaging studies of current flow in 3D-TI over a wide temperature range. Motivated by the above issues, we image the current flow in a Bi_2_Se_3_ from 15 upto 290 K, using the high sensitivity magneto-optical self-field imaging technique. We use this current imaging technique to study the nature of surface and bulk current distribution TI thin film and single crystal. We also employ bulk electrical transport measurements to understand our results. Notably Bi_2_Se_3_ thin film do not show uniform 2D sheet current flow, rather the flow is non-uniform sheet current. High current density flow is present along the film edges while a lower finite current density is present in the central regions of the film surface, away from the edges. Unlike the thin film, in Bi_2_Se_3_ single crystal at low *T* we readily observe highly uniform topological 2D sheet current flow associated with 2D surface state in 3D TI. We determine the sheet current thickness to be ~ 3.6 nm. With increasing temperature above 70 K, the uniform 2D conducting sheet disintegrates into smaller patches with high and low current density (*J*) distribution. Such imaging of uniform 2D sheet current flow in 3D TI single crystal and tracking its evolution as a function of temperature, to the best of our knowledge hasn’t been shown before. With decreasing *T*, the surface area fraction of the crystal with high *J* decreases rapidly above 70 K. The temperature dependence of the high *J* area fraction above 70 K follows the form, $${\raise0.7ex\hbox{$1$} \!\mathord{\left/ {\vphantom {1 {|T - 70|^{0.35} }}}\right.\kern-\nulldelimiterspace} \!\lower0.7ex\hbox{${|T - 70|^{0.35} }$}}$$.

## Results

### Transport measurement

**Figure 1:**
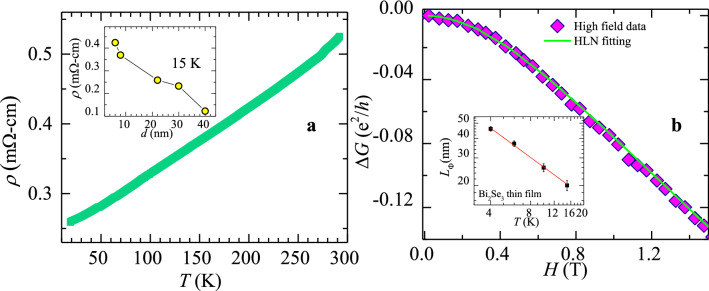
Magneto-transport properties of Bi_2_Se_3_ thin film. (**a**) Shows the temperature dependence of the resistivity of Bi_2_Se_3_ thin film. Inset figure shows the thickness dependence of resistivity at 15 K. (**b**) Figure shows the magneto-conductance in units of (*e*^2^/*h*), at 15 K. Green solid line shows fitting of the data to HLN equation (see text for details). Inset figure shows the behaviour of the electron phase coherence length, *L*_Φ_, as function of *T* on a log–log scale. The red line shows the linear behaviour (see text for details).

We grow epitaxial thin films of Bi_2_Se_3_ of dimensions 2.1 mm × 2.1 mm × 30 nm on STO (111) substrates by RF sputtering (see method and section I of supplementary for characterization details). Note the thickness of our film (30 nm) is greater than the threshold thickness of 5 nm for Bi_2_Se_3_, below which the top and bottom topological surface states hybridize, producing a trivial insulator^29^. The Fig. 1a shows a metallic behaviour of bulk resistivity (*ρ*) of the film measured as a function of increasing temperature (*T*). The unavoidable presence of intrinsic defects, like Se vacancies which electron dope the Bi_2_Se_3_ film^23,27^ lead to metallic conductivity in Bi_2_Se_3_. Inset of Fig. 1a shows a rapid decrease of *ρ* because with increasing film thickness (*d*). As Se vacancies concentration increases with increasing *d,* the doped charge (electron) concentration in the film also increases, resulting in a decrease in the *ρ*.

In zero applied magnetic field (*H*), spin momentum locking of Dirac electrons in the topological conducting surface state results in reduced back-scattering from disorder sites and weak anti-localization (WAL) effect. In TI’s at the special Dirac point in the energy-momentum dispersion spectrum of the surface states, the momentum states (+ *k* and − *k*) are doubly degenerate. Application of *H* lifts this degeneracy. At *H* = 0 the conducting surface states in TI have Berry phase (*ϕ*) = π. Breaking the time reversal symmetry (TRSB) with *H* ≠ 0 results in a decreasing *ϕ* with increasing *H*, and hence the conductance of the TI also decreases, i.e., Δ*G* (*H* ≠ 0) = *G*(*H*)–*G*(0) < 0. A combination of WAL along with TRSB effect gives rise to an inverted cusp feature in Δ*G*(*H*) for a TI. Figure 1b shows one half of the inverted cusp in Δ*G*(*H*) for our Bi_2_Se_3_ film, for *H* > 0. Figure 1b shows Δ*G* (*H*, 15 K) data, best fits the Hikami, Larkin and Nagaoka (HLN) model^30^, viz., $\Delta G = - \alpha \frac{{e^{2} }}{{2\pi^{2} \hbar }}\left[ {\ln \left( {\frac{{B_{\Phi } }}{H}} \right) - \psi \left( {\frac{1}{2} + \frac{{B_{\Phi } }}{H}} \right)} \right]$, where $\psi$ is digamma function, *e* is electronic charge, *α* = 0.89 (± 0.02) and $B_{\Phi }$ = 0.4 ± 0.01 T are the best fit parameters at 15 K. The phase coherence length of the electron in the TI is, $L_{\Phi } = \sqrt {\frac{\hbar }{{4eB_{\Phi } }}}$ = 48 (± 1.5) nm at 4 K which decreases to $L_{\Phi }$ ~ 20 nm at 15 K for the TI films (see inset of Fig. 1b). The typical reported $L_{\Phi }$ values are, about 100 nm for Bi_2_Se_3_ thin films grown with MOCVD and about 65 nm for sputtered films^31,32^. The inset of Fig. 1b shows a linear behaviour in a log–log plot of the phase coherence length $L_{\Phi }$ versus *T*, viz., $L_{\Phi } \propto T^{ - \lambda }$, where *λ* = 0.62 ± 0.02. The value of *α* is a measure of the effective number of conduction channels in the TI with *α* = 1/2 for each conduction channel^30^. From the fit at 4 K we get *α* close to 0.5, signifying conduction via Dirac electrons in the TI's surface. At 15 K, *α* increases towards 1 suggesting that additional conduction channels are present in the TI film.

### MOI_SF_ of Bi_2_Se_3_ thin film

**Figure 2:**
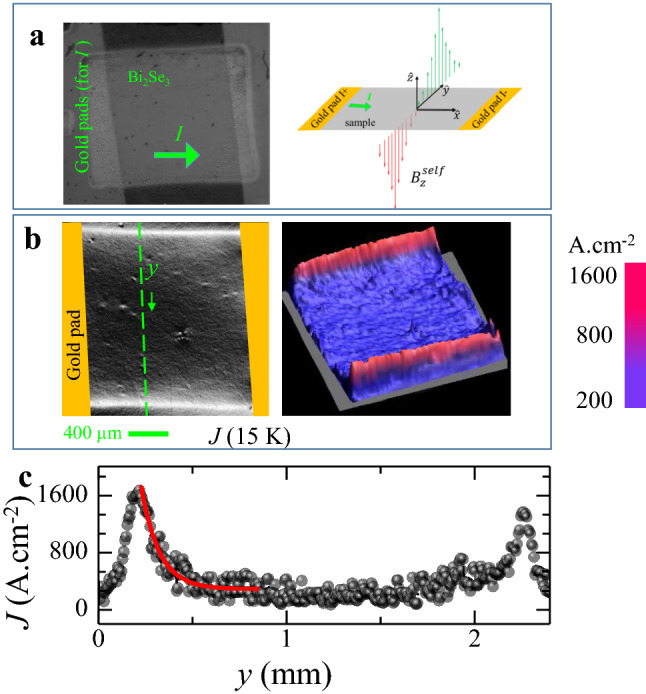
Edge current of thin film. (**a**) Figure shows an optical image of Bi_2_Se_3_ film showing the Cr/Au electrical pads sputtered on the film surface. Adjoining image is a schematic representation of the behaviour of the *z*-component of the self-field distribution. (**b**) Shows as gray-scale images and 3D map of the *J* (*x, y*) distribution across the sample surface at 15 K. (**c**) The figure shows the *J* (*y*) profiles (measured along the dashed green line in **b**) at 15 K. The red solid line is a fit to the data using $J = J_{0} \exp ( - \frac{{y - y_{0} }}{\delta }) + J_{b}$, where *J*_0_ = 1.6 × 10^3^ A/cm^2^, *J*_*b*_ = 260 A/cm^2^, *y*_*0*_ = 0.22 mm and *δ* = 96.70 μm.

We visualize current flow across the TI film using the self-field magneto-optical imaging technique (MOI_SF_) (see methods), which has been used to visualize current distribution in superconductors^33–35^. Briefly, MOI_SF_ technique involves high sensitivity spatial mapping of the average Faraday rotation at every location on the sample. The rotation angle relates to the self-field distribution, $B_{z}^{self} (x,y)$ (where (*x*, *y*) are the co-ordinates on the sample plane and *z* is perpendicular to it) generated by the current (*I*) sent across a sample (crystal/film). Figure 2a illustrates the schematic of $B_{z}^{self} (y)$ behaviour across the sample produced by the current. In the schematic, the $B_{z}^{self} (y)$ is shown along an imaginary line (along the y axis) drawn across the sample surface when current flows along the $\hat{x}$ direction. The length of the red and green vertical arrows schematically represents the variation in negative and positive $B_{z}^{self}$ values along *y*, respectively.

Using a numerical inversion scheme^36^, from the measured $B_{z}^{self} (x,y)$ we deduce the *J*_*x*_ (*x, y*) and *J*_*y*_ (*x, y*) components of the current density *J* (*x*, *y*) distributed over the sample surface. The direction of *J*_*x*_ and *J*_*y*_ are along and perpendicular to the applied current direction, respectively. In Fig. 2 we show only the magnitude of $J\left( {x,y} \right) = \sqrt {J_{x}^{2} \left( {x,y} \right) + J_{y}^{2} \left( {x,y} \right)}$. The overall direction of *J* is determined by the current sent into the TI sample. We calibrate the resultant *J* (*x, y*) map using the known current density in the Cr/Au contact pads. Figure 2a is an optical image of Bi_2_Se_3_ thin film with Cr/Au contact pads. Figure 2b shows at 15 K, in a gray scale image the *J* (*x, y*) distribution with *I* = 35 mA sent across the 30 nm thick Bi_2_Se_3_ film. The strong bright contrast represents high *J* at the film edges. At first glance the images suggest that current flow is through 1D wire like conducting edge states in a 3D TI. However, this is not possible as in 3D TI material such as ours has topological 2D high conducting surface states. Hence in 3D TI material, one should observe 2D surface sheet current flow instead of 1D edge current flow (which occurs only in 2D TI’s). A closer examination of the *J* (*y*) profile in Fig. 2c (measured along the green dashed line in Fig. 2b) shows existence of large *J* ~ 1600 A.cm^-2^ at the film edge which exponentially decays (see red fitted curve, in Fig. 2c) with a decay length ~ 96 μm. This decay length is substantially larger than $L_{\Phi }$ ~ 20 nm (15 K) (see inset of Fig. 1b). Hence the current at the edges isn’t a pure one-dimensional wire like edge current rather it is sufficiently broad. Note that the *J* in the central regions of the film away from the edges, isn’t zero, rather its ~ 200 A.cm^-2^. Thus, the edge *J* also doesn’t decay down to zero over nm length scales, as expected of purely one-dimensional edge current. Hence, the current flow in this 3D TI film isn’t via uniform 2D sheets rather it is via a non-uniform current sheet distributed over the film surface. This non uniform current distribution on the film surface has large *J* peaks near film edges with low but nonzero *J* in the central regions of the film. The 3D map of *J* (*x*, *y*) in Fig. 2b confirms that the above feature isn’t present at a few locations in the film but is uniformly seen across the entire film, viz., a uniformly high *J* at film edges which exponentially reduces to a smaller *J* in the film bulk.

### MOI_SF_ of Bi_2_Se_3_ single crystal

**Figure 3:**
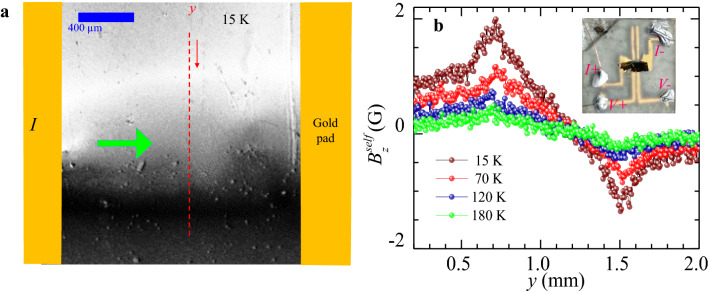
Imaging of the self-field distribution on Bi_2_Se_3_ single crystal. (**a**) Figure shows self-field magneto optical image representing *z*- component of self-field in the (*x*, *y*) plane, viz, $B_{z}^{self} (x,y)$ measured at 15 K. The green arrow represents the direction of the current (*I*) sent into the sample from the pads. (**b**) Figure shows $B_{z}^{self} (y)$ measured along the red dashed line in Fig. 3a at 15 K, 70 K, 120 K, and 180 K. Inset figure shows the optical image of the Bi_2_Se_3_ single crystal stuck on MgO substrate.

**Figure 4:**
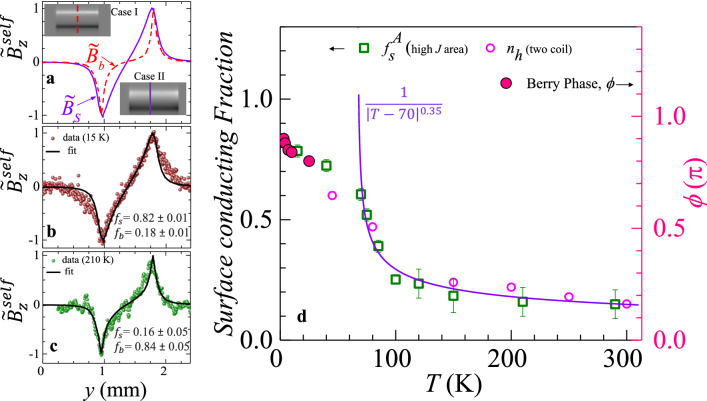
Analysis of self-magnetic field profile and fraction of surface contribution of electrical conductivity. (**a**) Figure shows the simulated magnetic field profiles $$\tilde{B}_{z}^{self} (y)$$ for bulk (case I) and surface (case II) currents distributed in the crystal. Upper and lower panels show as grey scale images, the simulated self-field distribution across the crystal, for the case I and II. Figures (**b**) and (**c**) show the behaviour of measured $$\tilde{B}_{z}^{self} (y)$$ and the Eq. 1 fit to the measured $$\tilde{B}_{z}^{self} (y)$$ data at 15 K, and 210 K, respectively. (**d**) The Berry phase ($${\raise0.7ex\hbox{$\phi $} \!\mathord{\left/ {\vphantom {\phi \pi }}\right.\kern-\nulldelimiterspace} \!\lower0.7ex\hbox{$\pi $}}$$) versus temperature (pink filled circles) calculated from the SdH oscillation observed on the magneto-resistance measurements on the crystal (see section II of supplementary) is shown on the right vertical axis. The green square symbol shows the area fraction of the high current density region ($$f_{s}^{A}$$) as a function of temperature (see text for details). Circle symbols represents the fraction of high conducting surface state ($$n_{h}$$) measured as a function of temperature, determined using the two-coil mutual inductance technique (reproduced from Fig. 5(a) of Ref.^28^). The violet solid line is the functional form of $${\raise0.7ex\hbox{$1$} \!\mathord{\left/ {\vphantom {1 {|T - 70|^{0.35 \pm 0.02} }}}\right.\kern-\nulldelimiterspace} \!\lower0.7ex\hbox{${|T - 70|^{0.35 \pm 0.02} }$}}.$$

Here we investigate electrical transport in Bi_2_Se_3_ single crystal. Supplementary section II, shows Shubnikov de Has (SdH) oscillations (between 4 K up to 25 K). The SdH oscillations show the presence of high conducting surface states present in this crystal. In the section II of supplementary, we show that the SdH is not due to the presence of a trivial two dimensional electron gas in Bi_2_Se_3_ but due to high conducting topological surface states located in the bulk gap of the material. The SdH fit gives the value of the parameter *ϕ* , viz., the calculated Berry phase to be ~ π. This value is expected for high conducting topological Dirac electrons present on the surface of TIs’. We image the current distribution in this single crystal of Bi_2_Se_3_ using MOI_SF_ technique. Figure 3a shows in gray scale the self-field $B_{z}^{self} (x,y)$ distribution across a Bi_2_Se_3_ single crystal at 15 K with *I* = 35 mA (note that if *I* were to distribute uniformly across the crystal cross section then, it corresponds to a current density of 194 A/cm^2^ for our crystal). The white and black contrast in Fig. 3a corresponds to $B_{z}^{self} (x,y)$ pointing either out of or into the plane of the figure (see section III, shows a linear increase of $B_{z}^{self}$ with *I*). Figure 3b shows the $B_{z}^{self} (y)$ profile, measured at different *T* along the red dashed line in Fig. 3a. At low *T* (15 K) the peaks in $B_{z}^{self} (x,y)$ are sharp, however as *T* increases the profile gets rounded off with a decrease in $B_{z}^{self}$ values. To understand the changes in the $B_{z}^{self} (y)$ profiles with *T* in Fig. 3b, we use COMSOL Multiphysics software to simulate field profile for 35 mA current sent through a 1.6 mm × 0.9 mm × 0.02 mm conductor (dimension identical to our crystal) for two different cases of current flow: Case I: Uniform cross-sectional current flow, viz., the $B_{z}^{self} (y)$ generated by a current which is uniformly distributed across the sample cross-section (with *J* = 194 A/cm^2^) and Case II: Surface current flow, viz., $B_{z}^{self} (y)$ produced by only two 2D surface current sheets (with *J* = 0.195 A/cm) on the top and bottom surfaces of the crystal and zero current in the sample bulk. Using finite current element analysis and the Biot-Savart’s law, we calculate the net $B_{z}^{self}$ generated by the current distributions in cases I and II. Grey scale images in the upper and lower panel of Fig. 4a show the simulated $B_{z}^{self} (x,y)$ distribution for (case I) and 2D surface sheet current (case II), respectively. Figure 4a compares the results for two cases, using normalized $\tilde{B}_{z}^{self} (y) = B_{z}^{self} (y)/\max \left\{ {B_{z}^{self} } \right\}$ for comparing the results. The $\tilde{B}_{z}^{self} (y)$ profiles for, the 2D surface current sheet (case II) and the bulk current (case I) are $\tilde{B}_{s}^{{}} (y)$ and $\tilde{B}_{b}^{{}} (y)$ respectively (see Fig. 4a). We convert the measured $B_{z}^{self} (y)$ profiles measured at 15 K and 210 K into $\tilde{B}_{z}^{self} (y)$ (cf. Figures 4b,c, respectively) and fit them with Eq. (1),

1 $$ \tilde{B}_{z}^{self} (y,T) = f_{s} \left( T \right)\tilde{B}_{s}^{{}} (y) + f_{b} \left( T \right)\tilde{B}_{b}^{{}} (y) $$

where $f_{s} (T)$ and $f_{b} (T)$ are the fraction of current flowing through the crystal 2D surfaces and crystal bulk respectively, and they obey $f_{s} (T) + f_{b} (T) = 1$. Figure 4b shows the $\tilde{B}_{z}^{self} (y)$ profile at 15 K best fits Eq. (1) (black solid line) with $f_{s} (T)$ ${\mathrm{f}}_{\mathrm{s}}\left(\mathrm{T}\right)$= 0.82 and $f_{b} (T)$ ${\mathrm{f}}_{\mathrm{b}}\left(\mathrm{T}\right)$= 0.18. Thus, at low *T,* the higher $f_{s}$ value suggests a dominant 2D sheet current flow along the crystal surface with a small fraction ($f_{b}$) of current flowing through the crystal bulk. It is important to note that our crystal thickness is well above the direct coupling limit (5 nm) of Bi_2_Se_3_. Hence, the observed sheet current comprises topological Dirac surface electrons in Bi_2_Se_3_ (recall the SdH oscillations observed, see section II). Compared to 15 K, the fit to Fig. 4c at 210 K gives $f_{s} (T)$ = 0.16, $f_{b} (T)$ = 0.84, viz., at high *T* the fraction of current flowing through the crystal bulk significantly increases compared to that at lower *T*. This shows that with varying *T* there is a transformation from surface dominated to bulk dominated electrical transport.

**Figure 5:**
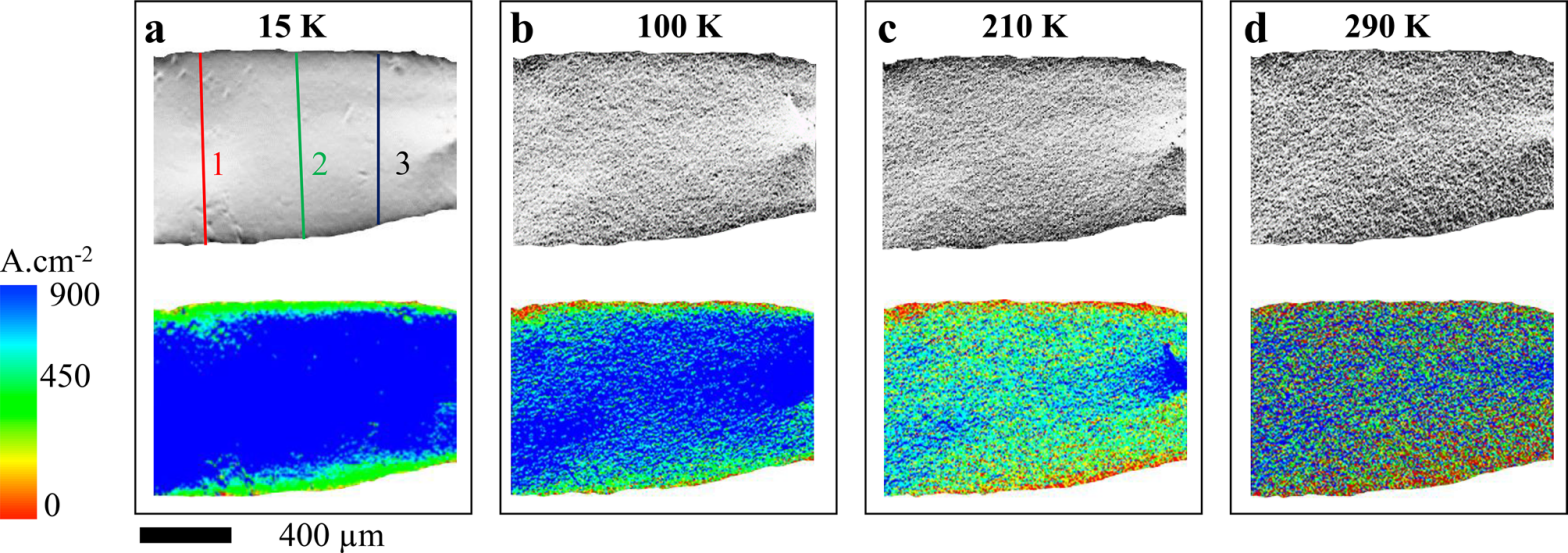
The evolution of *J* (*x, y*) distributions. Shows the *J* (*x, y*) distributions in grayscale and RGB scale inside the sample at (**a**) 15 K, (**b**) 100 K, (**c**) 210 K and (**d**) 290 K (see text for details).

In Fig. 5a–d we show the *J* (*x, y*) distribution over the crystal surface at 15 K, 100 K, 210 K, and 290 K (determined from the $B_{z}^{self} (x,y)$ distributions), as grey-scale images and coloured maps, respectively. Consistent with the inference drawn from the above discussions at 15 K, we see a uniform, high *J* (~ 900 A/cm^2^) two-dimensional (2D) current sheet on the crystal surface. This is in direct contrast to the non-uniform current distribution we had seen earlier for the Bi_2_Se_3_ thin film at 15 K. Our imaging in the single crystal confirms 2D high conducting surface states associated with a 3D TI material present at low *T* in the crystal, a feature which has not been imaged before. Note all prior imaging studies in thin films only saw one- dimensional wire like edge current channels. With increasing *T*, the uniform, 2D high *J* (≡ 770–900 A/cm^2^) surface sheet current in the crystal, disintegrates into low *J* (greenish and light blue, *J* ≤ 450 A/cm^2^) and high *J* (dark blue) regions (Fig. 5b–d). The onset of this inhomogeneous *J* distribution in the current images sets in from 70 K onwards where there is an increase in the distribution of current into the crystal bulk. The smallest high *J* features at 210 K are ~ 10 × 10 μm^2^. These are above the spatial resolution of our magneto-optical setup of 0.8 μm. Hence, we are not seeing any resolution limited features. We would like to mention that grainy feature that develops in the current distribution at high *T* (see Fig. 5 at 210 K) is also not due to noise in the captured images. Had the graininess been an experimental artefact due to noise in the images, then they should be present outside the sample as well as inside the sample. In the supplementary information section IV, by analysing features in the zoomed in portions of the image we explicitly show the absence of any of these grainy features either in the raw or the coloured images outside the sample boundaries. We also show in supplementary section IV that graininess is present both in the current distribution and the self-field images at 210 K (it is absent in the 15 K images). Thus, the development of graininess is not an artifact of numerical inversion algorithm used to obtain the current distribution from the measured self field images. We use images like those in Fig. 5, to measure the area of the high *J* (dark blue regions ≡ 770–900 A/cm^2^) regions at different *T* and determine $f_{s}^{A}$ = [total area of high *J* (dark blue regions) regions]/[crystals top surface area]. Figure 4d shows $f_{s}^{A} (T)$ abruptly decreases beyond 70 K, suggesting a transformation from surface to bulk dominated electrical transport. Above, 200 K the contribution from surface sheet current flow substantially reduces and the contribution to conduction from the crystal bulk dominates. Earlier studies^27,28^ showed that thermal activation of the charges doped in the bulk of Bi_2_Se_3_ crystal because of Se vacancies, results in a thermal activated behaviour of electrical conductivity above 70 K (also see section V of supplementary). To validate these estimates, Fig. 4d shows a close match between the values of high conducting surface fraction ($n_{h} (T)$) determined using another technique, viz., two coil measurements^28^ with the $f_{s}^{A} (T)$ values. A pure Dirac electron in 3D TI has a Berry phase (*ϕ*) = π. Figure 4d (pink filled circles) shows that in the crystal at low *T* (below 25 K), the (*ϕ*/π) value (determined from SdH fit, section II) is close to 1 and its *T* dependency is identical to that of $f_{s}^{A} \left( T \right)$. The comparison in Fig. 4d confirms that at low *T* the fluid of topological Dirac electrons uniformly covers the maximum surface area of the crystal. With the bulk conduction increasing from 70 K, consequently the 2D conducting sheet disintegrates into patches. The smaller dark blue patches which contain the high *J* (770–900 A/cm^2^) topological Dirac surface electron fluid are surrounded by normal electron fluid from bulk state which has a lower *J* (≤ 450 A/cm^2^). With increasing *T* above 70 K, the normal electron contribution arises in the TI because of an increasing bulk contribution to the electrical conduction. From the *J* (*x, y*) distribution at 15 K (Fig. 5a) we estimate the average effective thickness (*d*_*eff*_) of the topological conducting surface current sheet. We determine a quantity *K* by integrating *J* along each of the solid lines marked 1, 2, and, 3 in Fig. 5a, viz., $K_{1,2,3} = \int\limits_{1,2,3} {J \cdot dl}$, where *dl* is the length element along the line. Using, $K \cdot (2d_{eff} )\sim I$ for each of the lines (1, 2, 3), where *I* = 35 mA (factor two is for currents distributed along the top and bottom surface sheets in the TI) we get an average *d*_*eff*_ ~ 3.6 ± 1.0 nm. This value we have determined for the single crystal is close to the approximately 3 nm thickness of the topological surface state found in thin films of TI^37,38^.

## Discussion

In real 3D TI material like Bi_2_Se_3_ at low *T* the topological Dirac electron present on the surface of the material dominates in conduction. However, as the TI material gets electron doped via the Se vacancies, hence a conventional fluid of electrons appears in the material bulk at finite temperatures. Figure 4d show the surface fraction decreases with temperature but it has an abrupt break in curvature of the $f_{s}^{A} (T)$ close to 70 K. Figure 4d shows that for *T* > 70 K, the temperature dependence of $f_{s}^{A}$ is, $f_{s}^{A} \left( T \right)$ $\propto \frac{1}{{|T - 70|^{0.35 \pm 0.02} }}$. Recall our discussion of Fig. 5 had shown that near 70 K, coinciding with the onset of current distributing through the crystal bulk, the current flow transforms from a uniform 2D sheet current flow into patches of high and low *J*. Earlier studies^12,25^ show that the thermal activation energy of the charges doped in the bulk of Bi_2_Se_3_ crystal because of Se vacancies, is around 70 K [see section V, refs.^27,28^]. A recent room temperature STM study showed that nanoscale voids and defects in TI produce local variations in the topological surface state^39^. Our measurements show distinct features, viz., while ref.^39^ found local changes at the nanometer level which are temperature independent, we see high and low *J* macroscopic sized patches develop only after *T* crosses 70 K. At low *T* there is the topological Dirac electron fluid phase on the crystal surface which gives rise to uniform high *J*, 2D sheet current flow. Above 70 K a significant concentration of Se vacancy doped conventional electron fluid appears in the material bulk and we see the inhomogeneity in *J* distribution with patches of high *J* (770–900 A/cm^2^) region embedded in low bulk current density (≤ 450 A/cm^2^). We expect that, these two fluids have very different average energies as they occupy different bands in the material. With increasing *T*, especially above 70 K, the two fluids interact strongly. While we do not yet understand the nature of the complex interactions.

In summary, our study of Bi_2_Se_3_ single crystal at low *T* shows 2D like uniform surface state with high *J*. With increasing *T* above 70 K, the 2D like surface state with uniform high *J* sheet breaks up into smaller regions with high and low *J* distribution. Thermally activated delocalization doped charges generated by Se vacancies in the 3D TI bulk triggers this transformation. Future studies need to explore the quantum equivalent of this transition in 3D-TI. Such studies we hope will have a deep impact on our fundamental understanding of the properties of 3D TI materials and their technological applications.

## Material and methods

### Material

We study a single crystal of Bi_2_Se_3_ prepared by slow cooling of stoichiometric melts of high purity Bismuth (Bi) and Selenium (Se) powders. The crystal has dimensions of 1.9 mm × 0.9 mm × 0.02 mm. We have already investigated crystals from the same batch earlier using electrical transport^24^ and two-coil mutual inductance techniques^27,28^. Mechanical exfoliation gave freshly cleaved flat surface of the Bi_2_Se_3_ crystal. The Cr (5 nm) /Au (50 nm) electrical contact pads are DC sputtered on to the crystal / film surface. The four-probe resistance ~ 10 mΩ (300 K). We grew epitaxial thin films of Bi_2_Se_3_ of dimensions 2.1 mm × 2.1 mm × 30 nm on STO (111) substrates by RF sputtering (see section I of supplementary for thin film characterization). The SdH oscillations observed in our single crystal (see section II confirms that electrical conduction is predominantly via the topological surface states for *T* < 25 K).

### Magneto conductance measurement

We measured the magneto-conductance of our Bi_2_Se_3_ thin film using four probe geometry. We carried out high magnetic field transport measurement (solid square symbols in Fig. 1b) between 22 mT up to 6 T in the Physical Property Measurement System (PPMS, Quantum Design, USA). Remnant field of ~ 20 mT in the superconducting magnet coils in the PPMS prevented sensitive low field conductivity measurements below 20 mT. We used a home-built copper coil electromagnet for low field transport measurements from 1 mT up to 20 mT to avoid the remnant field issues. By comparing data from the two setups at higher field measurements, we determined good data overlap collected from the two setups.

### SFI method

In conventional magneto-optic imaging (MOI) technique the magneto-optical intensity of the Faraday rotated light reflected from the sample is proportional to the local magnetic field *B*_*z*_ (*x, y*) distribution across the sample when placed in an external magnetic field. In MOI_SF_ the Andor iXon (electron multiplied) EMCCD camera records the spatial distribution of Faraday rotation at every pixel in a 512 × 512-pixel image of the sample. The spatial distribution of the Faraday rotation is proportional to the local field distribution across the sample. In our case current (*I*) sent through the sample generates the self-field (viz., the local magnetic field). In this technique we capture differential images by taking the difference of magneto-optical images with a positive current (*I* +) and negative (opposite) current direction (*I-*) flowing through the sample. The MOI_SF_ is an image which shows the distributions of the magneto-optical intensity, *I*_SF_ (*x, y*), which is proportional to self-field distributions $B_{z}^{self} (x,y)$ so, $MOI_{SF}$ = $\frac{1}{M}\sum\nolimits_{j = 1}^{M} {\left[ {\frac{1}{N}\sum\limits_{i = 1}^{N} {\left( {MOI_{i} (I + ) - MOI_{i} (I - )} \right)} } \right]}_{j}$, where the (*x, y*) is a co-ordinate of a pixel in the image. The MOI_SF_ images represents the self-field, $B_{z}^{self} (x,y)$ generated by current *I*. To improve the signal-to-noise ratio, *N* nos. we capture the images with positive (*I* +) and negative currents (*I-*) and take the difference *M* number of times (in our case *N* = *M* = 20). Using a numerical inversion scheme^36^ we transform the $B_{z}^{self} (x,y)$ map into the current map *J* (*x, y*).

## Supplementary Information

Supplementary Information.
