# Supplementary material for: Imaging current distribution in a topological insulator Bi2Se3 in the presence of competing surface and bulk contributions to conductivity
Source: Sci Rep. 2021 Apr 2;11:7445. doi: 10.1038/s41598-021-86706-0 (PMC8018954; doi:10.1038/s41598-021-86706-0)
Supplement: Supplementary file 1 — Supplementary Information. [file 41598_2021_86706_MOESM1_ESM.docx]

Supplementary Materials for

**Imaging current distribution in a topological insulator Bi_2_Se_3_ in the presence of competing surface and bulk contributions to conductivity**

Amit Jash^1^, Ankit Kumar^1^, Sayantan Ghosh^1^, A. Bharathi^2^, S. S. Banerjee^1*^

^1^Department of Physics, Indian Institute of Technology, Kanpur 208016, Uttar Pradesh, India

^2^UGC-DAE Consortium for Scientific Research, Kalpakkam-603104, India

corresponding author email: satyajit@iitk.ac.in.

**Section I: Bi_2_Se_3_ thin film:**

The Bi_2_Se_3_ thin film has been grown on SrTiO_3_(111) substrate by the RF magnetron sputtering. A commercially available stoichiometric Bi_2_Se_3_ target from ALB Materials Inc of high purity of 99.999% was used as the sputtering target. The distance between the substrate and the sputtering gun was 6.4 cm. The base pressure of the sputtering chamber was kept at 5 ×10^-6^ mBar, and 8 ×10^-3^ mBar argon gas (99.99% purity) pressure was maintained during deposition. The SrTiO_3_ substrate temperature was kept at 390 ^o^C before and during the deposition. Before deposition, the substrates were carefully cleaned with acetone, alcohol, de-ionized water and purged with nitrogen gas. The RF power was fixed at 30 watt and average growth rate of Bi_2_Se_3_ thin film was 1 nm/min, with film thickness between 5 nm to 40 nm. After deposition, the film was post-annealed in the sputtering chamber in argon environment at temperature 350 ^o^C for four hours. Figure below shows the XRD (X-ray diffraction) pattern only displaying (0 0 3n) direction Bragg peaks of the Bi_2_Se_3_ thin film due to the three-fold symmetry of the Bi_2_Se_3_ crystal structure, the same as reported in past. The XRD direction was carried out in gazing angle mode which avoids the Bragg peak from the SrTiO_3_ substrate. The direction peaks of (0 0 3n) indicates the rhombohedral structure and the thin film growth along the (111) direction. The sharp XRD peaks indicate the high quality, epitaxial nature of our films.


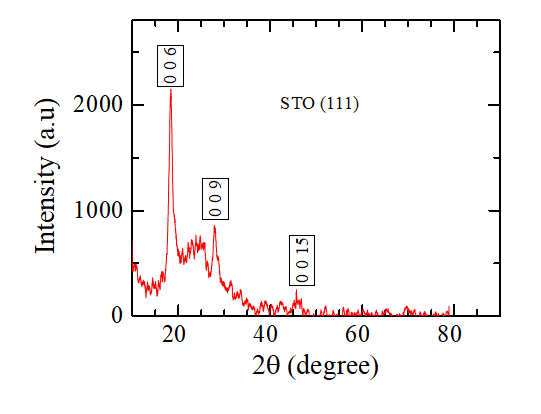


**Fig. S1.** **XRD characterization.** Figure shows the XRD peaks of Bi_2_Se_3_ thin film, grown on STO (111) substrate.

**Section II: Observation of SdH oscillation in the Bi_2_Se_3_ single crystal and location of the Fermi level within the bulk gap of the material:**

Figure (a) shows distinct SdH oscillation in longitudinal magneto-resistance (*ΔR_xx_* vs magnetic field (*B*)) measurements at 10 K using standard Van der Pauw geometry. This data is reproduced from A. Jash, et al., *Phys. Rev. Appl*. 12, 014056 (2019) (Ref. 26 in main MS). *ΔR_xx_* is calculated by subtracting from the experimental *R_xx_*(*B*) values with a polynomial fit to the data (*R_poly_* (*B*)), i.e, *ΔR_xx_* = *R_xx_*(*B*) - *R_poly_* (*B*). The polynomial form of *R* is $R_{poly}(B)=R_{0}+R_{1}B+R_{2}B^{2}$, where *R_0_* = 9.26×10^-3^ Ω, *R_1_* = -1.71×10^-6^ Ω.T^-1^, *R_2_*=2.91×10^-5^ Ω.T^-2^. Upper inset shows the variation of *R_xx_* as a function of magnetic field *B* measured at 10 K. Lower inset shows the SdH oscillation at different low temperature. We use Lifshitz-Kosevich (LK) equation: to analyse the SdH oscillation seen in our Bi_2_Se_3_ sample, where *F* and *β* are the fitting parameter. From the oscillation period (*F*) of the SdH oscillations seen in the transport data, the measured surface carrier density per area is found to be $n_{s}=(2.268\pm0.012) \times{10}^{12}$ cm^-2^, which corresponds to a Fermi wavevector for the 2D surface state to be $k_{F}=\sqrt{2\pi n_{s}}$= (0.0377±0.0027) Å^-1^. By placing the location of *k_F_* on the ARPES spectrum of Bi_2_Se_3_ (see *k_F_* marked by a vertical yellow line in the Fig. (b) which is Fig. 1 ARPES data of Bi_2_Se_3_ as published by M. Bianchi et al., *Nature Commun*. **1**, 128 (2010)), we see that the Fermi energy is approximately 30 meV above the Dirac point and about 100 meV below the bottom of the bulk conduction band (shown by green dash line). The position of the Fermi level suggests the carriers are from surface states and not from bulk bands. The charge carriers from bulk conduction do not contribute which suggests we do not have a non-topological two-dimension gas contributing to conductivity.

**Fig. S2. SdH oscillation.** (a) shows the SdH oscillation at 10 K. Upper inset shows the longitudinal resistance as function of magnetic field. Lower inset shows SdH oscillation at different temperature. (b) ARPES data of Bi_2_Se_3_, showing the position of the Fermi level.

The 2D carrier density corresponding to this Fermi wave vector (*k_F_*) is (2.268 ±0.012) ×10^12^ cm^-2^. Suppose, the origin of the SdH oscillation is assumed to be due to bulk electrons, the bulk carrier density should be $\frac{k_{F}^{2}}{4\pi c}=3.8\times10^{18}$cm^-3^ (per spin), where c=28.64 Å is the lattice spacing along c axis. However the bulk carrier density in the TI material as calculated from the hall coefficient, is $2.83\times10^{19}$ cm^-3^. This comparison shows that the features of our transport data at low *T* cannot be reconciled with bulk conduction electrons contributing to conductivity rather they are arising from 2D surface electrons.

Furthermore, from the LK fitting, one also obtains a value of the Berry phase as 0.86π at 4.2 K. For the two-dimensional Dirac fermion in the massless limit, the Berry phase is π which leads to a suppression of the back scattering from disorder due to destructive interference. In general, the Berry phase value being close to π is taken to suggests that SdH oscillation arises from the Dirac electron. From LK fitting of our data, shows the phase factor (Berry phase) is closer to π below 10 K (Fig. 4d) suggesting the SDH oscillation arises from Dirac surface state. The Berry phase values decreases from π with increasing temperature until above 30 K where bulk contribution to conductivity begins to dominate, the SdH oscillations are lost. This also suggests that at low *T* the SdH oscillations are related to 2D surface electrons contributing to electrical conductivity. Hence, the sheet current distributions at low temperature arises due to the topological surface current of Bi_2_Se_3_ single crystal (Fig. 5a in MS).

**Section III: Self-field due to different amplitude of current:**

Figure below shows the magnetic field profiles at 18 K for different applied current through the Bi_2_Se_3_ sample. Line scan is taken along the solid line to each $B_{z}^{self}$image at different current. The $B_{z}^{self}$ at 35 mA is 2.05 Oe (at the edge) which decreases to 0.60 Oe when 15 mA current is applied through the sample. The self-magnetic field strength increases linearly with the applied current which justifies the linearity response of driving current to the captured $B_{z}^{self}$distributions.

**Fig. S3. Linear behaviour of self-field with applied current.** Figure shows the profiles of $B_{z}^{self}$at 18 K for different applied current. Inset figure shows the self-field image at 18 K.

**Section IV: Spatial resolution and background signal**

At low *T*, a nearly uniform current density (Deep Blue, 770 A/cm^2^ - 900 A/cm^2^) is distributed all across the crystal surface (Fig 5a in the MS). As the temperature rises, above 70 K a significant concentration of Se vacancy doped conventional electron fluid appears in the material bulk and we see the inhomogeneity in *J* distribution with patches of high *J* (770 A/cm^2^ - 900 A/cm^2^) region embedded in low bulk current density (≤ 450 A/cm^2^). The minimum spatial resolution of our MOI setup is 0.8 micron. We can’t refer this as noise as the typical area of the blue region is much larger than the 1 micron size. Figures S4(a) and S4(b) show the grey and coloured scale image of current distribution of the sample with background at 15 K and 210 K. The graininess we see at 210 K is not related to any noise in the imaging. Infact if it was noise then we must see the same level of graininess outside the sample as well. We demonstrate this below.

Figures S4(a) and S4(b) below show at 15 K and 210 K, in both grey and coloured scale images of current distribution inside the sample along with a significant region outside the sample boundary. These regions have similar contrast for 15 K and 210 K while inside the sample the current distribution evidently becomes grainy with increasing *T*. At 210 K the graininess in the current distribution inside the sample is clearly very different from the smooth contrast outside the sample. Hence the graininess we observe inside the sample at 210 K is not a result of noise. Furthermore, the coloured images below show that, by using the same colour scale to colour the *J*, we see at 210 K the graininess in the images obtained outside the sample is completely different from that inside the sample. If the speckle feature was due to a noise, then the speckle feature should be seen outside the sample at both 15 K and also more importantly at 210 K. However, we do not observe this. Therefore, the high temperature feature we see in the current distribution is not due to noise. Outside the sample we never observe the generation of any blue coloured speckle feature.

We show below that scale and nature of inhomogeneity are completely different inside the sample compared to that outside the sample. It is clear that inside the sample the nature of the current distribution changes from a smoother and more uniform distribution at 15 K to a much grainer current distribution at 210 K. These changes inside the sample occur while the contrast outside the sample for 15 K and 210 K are almost the same. To supports our view further, we have zoomed into a portion of the image which contains both the sample and background, which is shown in Fig. S4(e) and Fig. S4(f). From these zoomed images (in both gray scale and coloured images), the differences in the graininess inside and outside the sample is very evident an undeniable. While the complex grainy structure of current distribution develops inside the sample at 210 K (Fig. S4(e)), the background signal (outside the sample) has few uncorrelated weak white dots. In the colour image, when we colour coded the current density (*J*) value to red colour from *J* range 0 to 350 A.cm^-2^, green colour from 300 A.cm^-2^ to 700 A.cm^-2^ and blue colour from 650 A.cm^-2^ to 900 A.cm^-2^, the background signal contrast further appears different from the sample spackles feature, shown in the zoomed image of Fig. S4(f).

In order to analyse the images further, figure S4(g) shows the average Fast Fourier Transform (FFT) of multiple line scans (intensity versus distance) taken inside the sample and line scans across the background (see zoomed in image (e) where the typical location of a line scan taken outside (dashed green line) and inside (solid yellow line) the sample are shown). In Fig. S4(g) the background FFT is featureless as is expected for noise in the imaging. Inside the sample we see there are peak structure in the sample FFT, which suggest the grainy features have a typical size. The distinction between noise present outside the sample and the grainy feature seen developing inside the sample due to onset of inhomogeneous current distribution with increasing *T*, is changed is clear.

**Fig. S4. Background signal at low and high temperature.** The grey scale image of the current density distribution in the sample at (a) 15 K and (b) 210 K. (c) and (d) show the current density (*J*) in the images (a) and (b) mapped onto to a colour scale shown beside fig. (e) is zoomed in portion of the red rectangular region in the image shown in (b). In this zoomed image the lower half of the image is inside the sample while the upper half is outside the sample. (f) is zoomed in portion of the red rectangular region in the image shown in (d). (g) FFT signal of the intensity versus distance measured along the line scans taken across the sample (along the green lines in fig.(e)) and along the lines outside the sample (along the yellow lines in fig.(e)).

In the figure below we show in Figs. 4R(a) and 4R(b) images of the current distribution across the sample at 15 K, and 210 K respectively. The images shown in figs. 4R(c) and 4R(d) are self-field images at 15 K and 210 K of the zoomed in portion of the sample region shown in figs. 4R(a) and 4R(b). We show the zoomed in portions of the sample in order to bring out the differences in images clear. It is clear from the zoomed in self-field image at 210 K (Fig. 4R(d)) that inhomogeneity is present in the raw self-field image. The degree of inhomogeneity at 210 K in the self-field is absent in the 15 K self-field image (Fig. 4R(c)). Compared to 210 K the self-field image at 15 K is uniform. Hence, the speckles like features appearing in the inverted current image at 210 K (Fig. 4R(b)) is not due to the numerical inversion technique of the self-magnetic field used to obtain the current density image

**Fig. R4. Raw self-field distribution.** (a) and (b) are the images of the current distribution across the sample at 15 K, and 210 K respectively. The images (c) and (d) are the self-field images at 15 K and 210 K of the zoomed in portion of the sample regions shown in (a) and (b). The difference in the degree of graininess of the images at 210 K compared to that at 15 K are clearly visible in the zoomed images.

**Section V: Thermally activated behaviour at 70 K.**


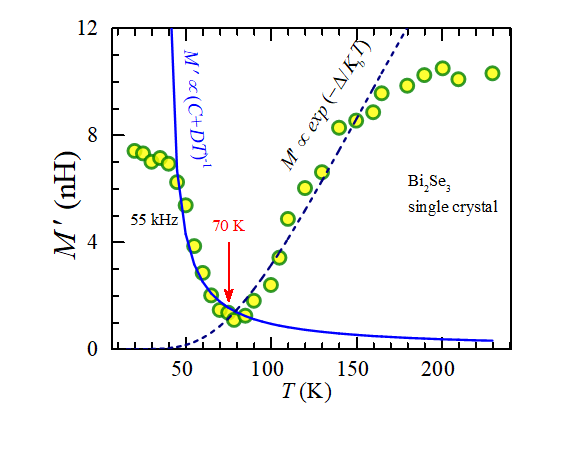


**Fig. S5. Thermally activated conduction from 70 K.** Shows the real part of mutual inductance ($M^{'}$) as function of temperature of Bi_2_Se_3_ single crystal. Surface and bulk conduction dominated regions are observed at temperature window.

The data presented above is measurement of inphase component of the mutual inductance $M'$ of a pickup coil setup. Briefly in the setup, between a primary excitation coil and a secondary pickup coil a Bi_2_Se_3_ single crystal is placed (for details see A. Jash, et al., *Phys. Rev. Appl*. 12, 014056 (2019) (Ref. 27 in main MS)). An AC current in the primary coil generates a magnetic field which induces a pickup voltage in the secondary coil. The two coils are coupled via the TI sample placed in between the two coils. As the flux linkage between the two coils is through the TI sample, hence the mutual inductance depends on the properties of the TI crystal. The inphase component of the pickup voltage $V^{'}=M^{'}\omega I_{primary}$ where *ω* is the angular frequency of the current in the primary coil, *I_primary_*. One can show that $V^{'}$(and hence also $\left( M^{'} \right))\propto\sigma$, where *σ* is the conductivity of the TI sample (see above reference). From $V'$ one can get a measure of $M'$.

We have identified the thermally activated type of conduction in the bulk of Bi_2_Se_3_ from 70 K onwards using our two-coil setup. Figure S5 shows the $M^{'}(T)$ behaviour at 55 kHz of Bi_2_Se_3_ single crystal (sample thickness 69 µm). Below 70 K the $M^{'}(T)$ data fits (solid blue line) to a form $\frac{1}{\left(C+DT\right)}$ , which corresponds to the *T* dependence of $\sigma$. $\sigma\propto\frac{1}{\left(C+DT\right)}$, is the typical temperature dependence of the topological conducting surface states in a TI (Ref.26 and reference there in). While above 70 K to 170 K the data is fitted (dashed line) which corresponds to bulk contribution to $\sigma$ of the form $\sigma\propto\exp\left(\frac{-\Delta}{K_{b}T}\right)$, Δ ~ 25.2 ± 1.25 meV, where Δ is an activation energy scale and$\sigma_{b0}$ is the high temperature conductance of the bulk state. Electron doping by Se vacancies generates a disorder band in the Bi_2_Se_3_ crystal. This disorder band lies Δ below the bulk conduction band minima in the bulk gap (~ 0.3 eV) of Bi_2_Se_3_. As shown in the main MS, concomitant with the onset of conduction in the bulk beyond 70 K, patches of high and low current density region proliferate the uniform conducting, topological uniform current carrying sheet on the surface of Bi_2_Se_3_ single crystal starts (see fig. 5 in main MS).

From this figure, note that at low temperatures below 40 K, the weak *T* dependence suggests *C* << *D*, i.e.., the electron-phonon interaction contribution to temperature dependence of conductivity (and hence to $M^{'}$) is weak and so also is the thermally activated component (see black dashed line below 70 K). The solid blue line fit to the data to 1/[*C+DT*], shows that at high *T* the electron-phonon interaction contribution to temperature dependence of conductivity (and hence to $M^{'}$) is very weak compared to the contribution from thermally activated behaviour.
